# Effects and Moderators of Exercise on Sarcopenic Components in Sarcopenic Elderly: A Systematic Review and Meta-Analysis

**DOI:** 10.3389/fmed.2021.649748

**Published:** 2021-05-19

**Authors:** Yanjie Zhang, Liye Zou, Si-Tong Chen, Jun Hyun Bae, Dae Young Kim, Xiaolei Liu, Wook Song

**Affiliations:** ^1^Health and Exercise Science Laboratory, Institute of Sports Science, Seoul National University, Seoul, South Korea; ^2^Physical Education Unit, School of Humanities and Social Science, The Chinese University of Hong Kong–Shenzhen, Shenzhen, China; ^3^Exercise Psychophysiology Laboratory, Institute of KEEP Collaborative Innovation and Mental, School of Psychology, Shenzhen University, Shenzhen, China; ^4^Institute for Health and Sport, Victoria University, Melbourne, VIC, Australia; ^5^Chinese Traditional Regimen Exercise Intervention Research Center, Beijing Sport University, Beijing, China; ^6^Institute on Aging, Seoul National University, Seoul, South Korea

**Keywords:** physical exercise, muscle function, physical performance, sarcopenia, meta—analysis

## Abstract

**Background:** Sarcopenia is a muscle disease in loss of muscle strength, mass, and function associated with aging. Although protective effects of exercise on muscle mass and function are generally recognized, research findings in sarcopenic adults are inconsistent. It is necessary to conduct a systematic review to determine the effects of exercise on muscle strength, body composition, and physical performance in older adults with sarcopenia, and to examine the potential moderators including sociodemographic characteristics and exercise-related factors.

**Methods:** Six electronical academic databases (Medline, Embase, CINAHL, Scopus, Cochrane Library, and SPORTDiscus) were used to retrieve the eligible studies from inception to May 2020. Two reviewers independently selected and extracted the data from each included study, and effect sizes were calculated by employing random-effect models with 95% confidential interval (CI). The Physiotherapy Evidence Database (PEDro) scale was used to assess study quality.

**Results:** Seventeen studies (985 participants with sarcopenia, aged 67.6–86 years) were included in this review study. The meta-analytic results showed significant improvements in muscle strength [grip strength, SMD = 0.30, 95% CI (0.15, 0.45), *I*^2^ = 6%, *p* < 0.01; knee extension, SMD = 0.32, 95% CI (0.15, 0.50), *I*^2^ = 0%, *p* < 0.01; and chair and stand, SMD = 0.56, 95% CI (0.30, 0.81), *I*^2^ = 36%, *p* < 0.01], in physical performance [timed up and go, SMD = 0.74, 95% CI (0.48, 1.00), *I*^2^ = 0%, *p* < 0.01; and gait speed, SMD = 0.59, 95% CI (0.35, 0.82), *I*^2^ = 62%, *p* < 0.01], and in body composition [skeletal muscle mass index, SMD = 0.37, 95% CI (0.15, 0.58), *I*^2^ = 16%, *p* < 0.01; and appendicular skeletal muscle, SMD = 0.31, 95% CI (0.13, 0.49), *I*^2^ = 20%, *p* < 0.01]. However, there were no significant differences in other body composition (SMD = 0.20–0.36). Additionally, meta-regression revealed that the higher percent of female participants was significantly associated with improved gait speed (β = 0.0096, *p* = 0.03) and decreased skeletal muscle mass index (β = −0.0092, *p* = 0.01).

**Conclusions:** The current meta-analysis suggests that exercise is a beneficial therapy, which has protective effects for older adults with sarcopenia. Some beneficial effects may be moderated by gender and exercise intensity.

## Introduction

Aging-related health leads to many issues in the 21^st^ century. One of the major public health challenges is to preserve older adults' physical ability and quality of life, to achieve successful aging in the whole society (1). However, due to internal physiological changes in the human body, gradual declines in skeletal muscle strength, losses of muscle mass, and reductions in physical capacity are inevitable during the aging process. These reductions are commonly known as sarcopenia (2, 3). Despite no consistent diagnostic criteria for sarcopenia, the prevalence and harmfulness of sarcopenia in older adults have been shown to be ubiquitous. For example, a previous review study using the European Working Group on Sarcopenia in Older People (EWGSOP) optional definition found that the prevalence of sarcopeina was between 11 and 20% in old adults (4). Subsequently, a series of adverse health outcomes such as mental illness, physical limitations, fractures, poor therapeutic efficacy, poor quality of life, cancer, and even cachexia in adults, have been shown to be associated with the presence of a high-risk of sarcopenia (5–9). Therefore, to reduce the prevalence of sarcopenia, there is a growing interest in non-pharmacological treatments, such as physical exercise, in which researchers can determine the effects and design optimal interventional strategies.

A wealth of evidence supports that exercise, an important modified lifestyle factor, is feasible and efficacious in improving psychological outcomes (e.g., cognition, mood) in older adults (10–14). Moreover, the literature supports that exercise improves physiology-related muscle strength, muscle strength, and physical performance in older adults, and the associated studies are also growing rapidly (15–17). For example, the randomized study of Liu et al. consisting of older adults, found that physical exercise could elevate physical performance (18). A systematic review study examining the impact of resistance exercise showed that low-load exercise benefited the muscle strength and muscle mass in older adults (19). However, in the literature, health benefits of exercise in sarcopenic adults yields mixed findings. For example, a recently published systematic review including five randomized controlled trials (RCTs) suggested that resistant exercise can improve muscle strength, muscle quality, and muscle function in older adults with sarcopenia or dynapenia compared with the controlled group (20). Similarly, the systematic review of Beckwée et al. concluded that exercise contributed to improving muscle strength, muscle mass and physical performance of sarcopenic adults (21). Conversely, the work of Yoshimura et al. demonstrated that exercise had no significant effects on muscle strength, muscle mass, and physical performance in older adults with sarcopenia (22). A systematic review study including six studies indicated that exercise did not significantly increase muscle strength, muscle mass, and balance ability among sarcopenic adults (23). These discrepant research findings imply that more synthesized studies should confirm the roles of exercise on health outcomes in older adults with sarcopenia. In response to this, a meta-analysis based on multiple studies is necessary to demonstrate the effects of exercise on sarcopenia.

Additionally, it is important for sarcopenic older adults to have optimized exercise programs that can promote their physical health. A previous study has found that the exercise modalities (e.g., duration, intensity, and type) were not met to counteract sarcopenia (24). Denison and colleagues found that demographics (e.g., age and sex) can moderate the effects of exercise in older adults with sarcopenia (25). Considering rapid growth in the research field of exercise and sarcopenia, aggregating sufficient quality studies for meta-analysis cannot only make up the limitations across previous reviews but also comprehensively determine the effects of exercise and then examine the influences of moderating factors.

Therefore, the current study was conducted: 1) to determine the effects of exercise on muscle strength, physical performance, and body composition in older adults with sarcopenia and 2) to investigate whether the potential moderators including sociodemographic characteristics and exercise-related factors that influence the intervention effects, as these moderators have been found affecting the sarcopenia-related health outcomes (25).

## Methods

### Search Strategy

This systematic review was prospectively registered at PROSPERO (ID: CRD42020184130; https://www.crd.york.ac.uk/) and performed in line with the Preferred Reporting Items for Systematic Reviews and Meta-Analyses (PRISMA) guidelines (26). Articles were retrieved from six databases (Medline, Embase, CINAHL, Scopus, Cochrane Library, and SPORTDiscus) on May 2020. The following keywords were used: 1) “physical activity” OR “physical therapy” OR “aerobic exercise” OR “exercise^*^” OR “resistance training” OR “train^*^; AND 2) “sarcopenia” OR “sarcopenic” OR “dynapenia” OR “muscular atrophy” OR “muscular weight” OR “grip strength”; AND 3) “older adults” OR “aged” OR “elder^*^”; AND 4) “randomized controlled trials” OR “clinical trial” OR “random allocation.” To retrieve more eligible articles, manual searching was conducted from the bibliographies of the included studies.

### Inclusion and Exclusion Criteria

Articles were included if they met the following criteria: (i) Participants aged over 65 years were diagnosed as sarcopenia based on the definition of EWGSOP, Asia Working Group for Sarcopenia (AWGS) or other clinical diagnosis. (ii) The study was designed as an RCT. (iii) Exercise (e.g., aerobic exercise, resistance training, whole-body variation, or a combination of strength and aerobic exercise program) was used in the intervention group. (iv) No exercise intervention (e.g., usual care or waitlist) was given in the control group. (v) The outcomes were muscle strength, body composition (skeletal muscle mass index, appendicular muscle mass, lean mass, body fat, and fat-free mass), and physical performance (gait speed and timed up and go test). (vi) The study was published in the English language.

Exclusion criteria were: (i) *in vitro* with an animal trial, (ii) participants with sarcopenia obesity, (iii) insufficient information for calculating the effect size (ES), And (iv) case-study, observational studies, editorials, or review articles.

### Data Extraction and Quality Assessment

Detailed information was extracted from each study through a pre-created extraction table by two authors (YZ and LZ). The presenting information included the author and year of publication, study design, participants' characteristics, interventions, sarcopenia diagnostic criteria, assessment tool for body composition, outcomes, and safety.

Assessment of study quality was conducted using the Physiotherapy Evidence Database (PEDro) scale (27) by two authors (YZ and LZ). This assessment tool consists of 11 items: eligibility criteria, random allocation, concealed allocation, similar measures between groups at baseline, instructor blinding, assessor blinding, participant blinding, more than 85% dropout rate, intention-to-treat analysis, statistical comparison between groups, and ≥1 key outcome estimated. Each item is scored as 0 (absent) or 1 (present). The total score is in the 0–10 point range after summing scores of all items. The study quality was classified as excellent (9–10 points), good (6–8 points), fair (4–5 points), and poor (< 4 points).

### Statistical Analysis

The Comprehensive Meta-Analysis program (version 2.2) was used for analyzing the extracted data. Since all extracted outcome data were continuous variables with variability between studies, standard mean differences (SMDs) were used for representing the ESs by calculating the mean change from baseline to post-intervention for the intervention and control groups. If there were two exercise groups (or control group) in one study, we halved the number of participants in the control group (or exercise group), while the mean and SD were unchanged. The random effects model, which can avoid the high risk of false-positive results, (28) was used with 95% confidence interval (CI) in overall ESs estimated. According to the Cochrane handbook, the ES was classified as small (0.2–0.49), moderate (0.50–0.79), and large (≥0.8) (29). A positive ES value indicated that the results were more favorable to the intervention group, otherwise the control group. Study heterogeneity was evaluated using the *I*^2^ test, which was classified as three levels: low, moderate, and high heterogeneity with cutoff points (*I*^2^ = 25, *I*^2^ = 50, and *I*^2^ = 75%). We assessed the publication bias using Egger's regression test and Funnel plot. The Duval and Tweedie's trim and fill method was used to assess the potential impact of this publication bias. A two—side analysis with a significant level of 0.05 was used in all analyses.

Additionally, the continuous and categorical moderator analyses were performed using random effects model to investigate the influences of potential moderators on the overall ESs. Continuous moderators included mean age, exercise duration, and dose of exercise intervention (overall number of weeks of exercise × mean exercise frequency weekly × mean exercise time of each session in minutes). Potential categorical moderators included sex, diagnosis criteria, and exercise characteristics (exercise type, exercise frequency, exercise duration, exercise time, and exercise intensity). The coding of diagnosis criteria was categorized as the AWGS and EWGSOP. According to the classification of exercise prescription in a previous study (10), the type of exercise was coded as aerobic exercise, resistance exercise, and mixed exercises. Exercise frequency was coded as <3 and ≥3 times. Exercise duration was defined as ≤ 12 weeks (short term) and >12 weeks (long term) in this study. Exercise time was coded as < 45 min (short) and 45–60 min (medium). Exercise intensity was coded from light to vigorous intensity according to the American College of Sports Medicine (30). The control group was coded as active control (e.g., nutrition, health education) and passive control (e.g., waitlist).

## Results

### Search Results

**Figure 1:**
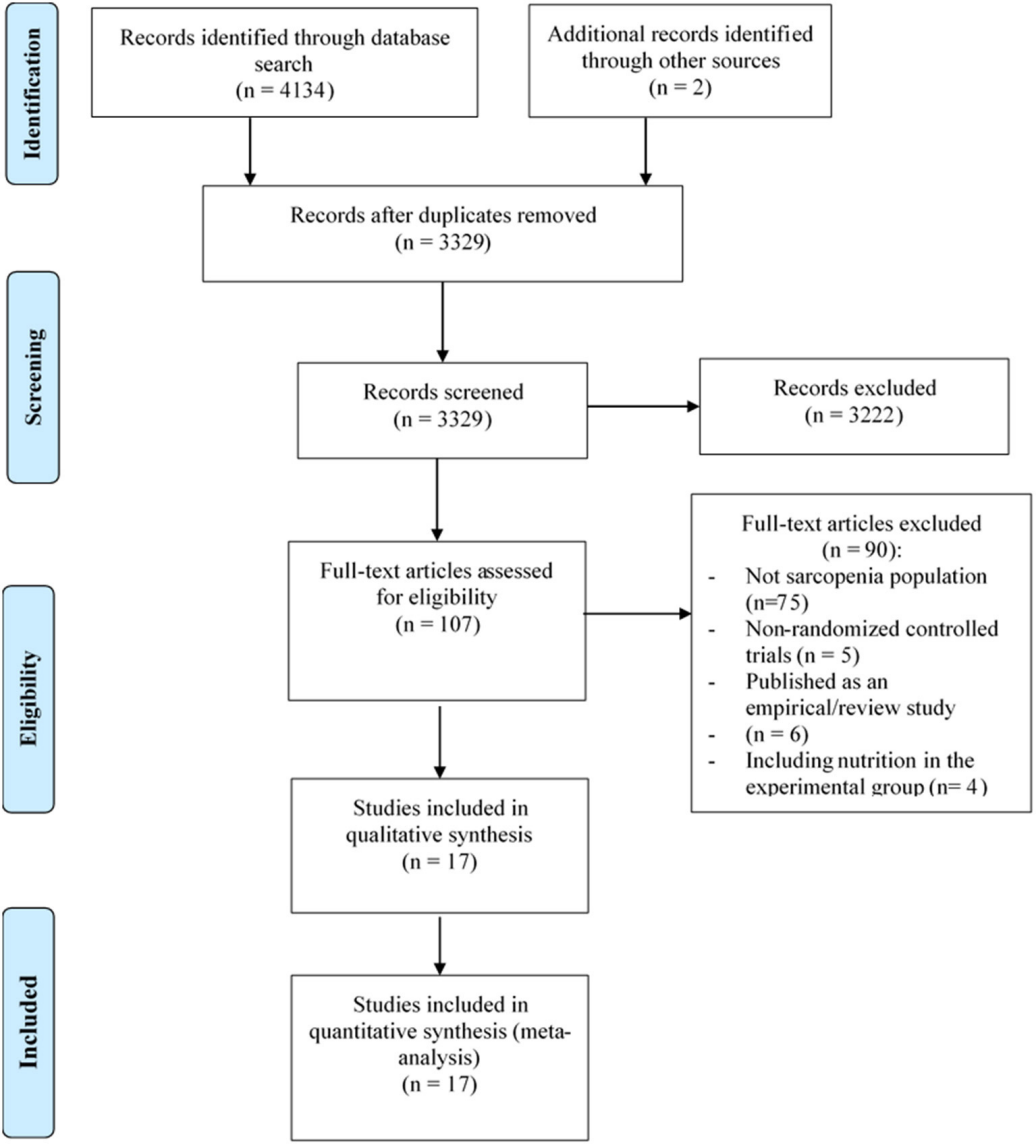
Process of study selection following the Preferred Reporting Items for Systematic Reviews and Meta-Analyses (PRISMA).

Figure 1 depicts the process of study selection of this review. A total of 4,136 studies were retrieved from the electronic databases, 807 of them were excluded because of duplicates, and 107 full-text articles were identified for further confirmation after screening the titles or abstracts. Subsequently, the remaining 107 studies were reviewed for eligibility through reading the full-texts. Finally, 17 studies (31–47) were considered as eligible studies that were included in the meta-analysis.

### Characteristics of Included Studies

The characteristics of the included 17 studies are summarized in Table 1. The included studies were published between 2012 and 2020, locating at 12 countries Australia (32), Spain (40), Korea (41), Germany (44), Iran (45), Japan (42, 43, 47), Italy (33), Malaysia (48), Austria (34), Greece (35), Sweden (36), and China (37, 39). A total of 985 participants with sarcopenia were included in the included 17 studies, and the mean age ranged from 67.6 to 86 years. Of note, the diagnostic sarcopenia was based on the two criteria, EWGSOP and AWGS. Eleven studies (31–33, 35, 37, 38, 40, 42, 43, 46, 47) used BIA, and six studies (34, 36, 39, 41, 44, 45) used dual-energy X-ray absorptiometry (DXA) to measure the body composition. A large proportion (74%) of females was evaluated in the included studies (7/17 studies were all females). Participants in the experimental group were given aerobic exercise, resistance exercise, or mixed exercises. These participants were concurrently given 30–60 min of exercise in each session, one to seven times per week for 8–36 weeks. Usual care or waitlist were provided in the control group. The outcomes in these 17 studies were grip strength, knee extension, chair and stand test, gait speed, timed up and go (TUG), appendicular skeletal muscle (ASM), skeletal muscle mass index (SMI), lean mass, body fat, and fat-free mass. There were no reports on side effects related to the exercises.

**Table 1:** Characteristics of randomized controlled trials included in the meta-analysis.

**Study/country**	**Participants/living status**	**Sample size Female (%)**	**Age (years)**	**Intervention(s)**		**Sarcopenia criteria**	**Assessment tool for body composition**	**Outcomes**	**Adverse effect**
				**Experiment group**	**Control group**				
Chen et al. (2018) (31)China	Sarcopenia residents of community dwelling	33 E = 17 C = 16 Female (100%)	67.5	2 × 60 min/week 8 weeks Resistance training	Waitlist	AWGS	BIA	Grip strength SMI ASM Body fat	No
Hassan et al. (2016) (32)Australia	Sarcopenia residents of nursing care facilities	41 E = 20 C = 21 Female (71%)	85.9	2 × 60 min/week 24 weeks Progressive resistance and balance training	Usual care	EWGSOP	BIA	Grip strength Gait speed SMI Lean mass Body fat	No
I Iranzo et al. (2018) (40)Spain	Sarcopenia residents in institution	28 E = 11 C = 17 Female (75%)	81.9	3 × 30–40 min/week 12 weeks Resistance training	Waitlist	EWGSOP	BIA	Grip strength Gait speed SMI	No
Jung et al. (2019) (41)Korea	Sarcopenia residents of community dwelling	26 E = 13 C = 13 Female (100%)	75	3 × 25–75 min/week 12weeks Resistance training and walking	Usual care + education	AWGS	DXA	Knee extension strength SMI Lean mass body fat	No
Kim et al. (2012) (42)Japan	Sarcopenia residents of community dwelling	117 E = 39 C1 = 39 C2 = 39 Female (100%)	79	2 × 60 min/week, 12 weeks Resistance training	C1: Nutrition C2: Health education	AWGS	BIA	Knee extension strength Gait speed ASM	No
Kim et al. (2013) (43)Japan	Sarcopenia residents of community dwelling	96 E = 32 C1 = 32 C2 = 32 Female (100%)	80	2 × 60 min/week, 12 weeks Resistance training	C1: Nutrition C2: Health education	AWGS	BIA	Grip strength Knee extension strength Gait speed TUG ASM	No
Lichtenberg et al. (2019) (44)Germany	Sarcopenia residents of community dwelling	43 E = 21 C = 22 Female (0%)	78.5	2 × 50 min/week 12 weeks Resistance training	Nutrition	EWGSOP	DXA	Grip strength Gait speed SMI	
Mafi et al. (2019) (45)Iran	Sarcopenia residents of community dwelling	47 E = 14 C1 = 17 C2 = 16 Female (0%)	68.5	3 × 60 min/week 8 weeks Resistance training	C1: Nutrition C2: Waitlist	EWGSOP	DXA	TUG ASM	No
Makizako et al. 2020 (46)Japan	Sarcopenia residents of community dwelling	72 E = 36 C = 36 Female (70.8%)	75	1 × 60 min/week 12 weeks Resistance training and aerobic exercise	C1: Waitlist	AWGS	BIA	Grip strength Chair and stand Gait speed TUG	No
Maruya et al. (2016) (47)Japan	Sarcopenia residents of community dwelling	52 E = 34 C = 18 Female (56%)	69	1 × 90 min/week, 24 weeks Walking and resistance training	Usual daily activity	AWGS	BIA	Grip strength Knee extension strength Gait speed SMI Body fat	No
Piastra et al.(2018) (33)Italy	Sarcopenia residents of community dwelling	72 E = 35 C = 37 Female (100%)	70	2 × 60 min/week, 36 weeks Resistance training	Postural activation	EWGSOP	BIA	Grip strength SMI ASM Lean mass	No
Strasser et al. (2018) (34)Austria	Sarcopenia residents of institution	33 E = 16 C = 17 Female (91%)	83	2 × 60 min/week 24 weeks Resistance training	Cognitive training	EWGSOP	DXA	SMI ASM	No
Tsekoura et al. (2018) (35) Greece	Sarcopenia residents of community dwelling	54 E1 = 18 E2 = 18 C = 18 Female (84%)	73	E1: 2 × 60 min/week, 12 weeks Resistance training and 3 × 30–35 min/week, walking E2: same E1, home therapeutic exercises	Health education	EWGSOP	BIA	Grip strength Knee extension strength Chair and stand Gait speed TUG SMI Fat-free mass	No
Vikberg et al. (2019) (36)Sweden	Sarcopenia residents of community dwelling	70 E = 36 C = 34 Female (54%)	70.5	3 × 45 min/week, 10 weeks Resistance training	Waitlist	EWGSOP	DXA	Grip strength Chair and stand TUG Gait speed SMI ASM Lean mass Fat mass	No
Wei et al. (2016) (37)China	Sarcopenia residents of community dwelling	40 E = 20 C = 20 Female (70%)	76	3 × 24 min/week 12 weeks Whole-body vibration training	Waitlist	EWGSOP	BIA	Knee extension strength TUG Chair and stand	No
Yamada et al. (2019) (38)Japan	Sarcopenia residents of community dwelling	84 E = 28 C1 = 28 C2 = 28 Female (63%)	83.9	2 × 30 min/week 12 weeks Resistance exercise	C1: nutrition C2: waitlist	AWGS	BIA	Grip strength Knee extension strength Chair and stand Gait speed ASM	
Zhu et al. (2019) (39)China	Sarcopenia residents of community dwelling	77 E = 40 C = 37 Female (75%)	73	2 × 45–60 min/ week 12 weeks Resistance training and aerobic exercise +1 time/week home exercise	Waitlist	AWGS	DXA	Grip strength Knee extension strength Chair stand test Gait speed ASM	No

*ASM, appendicular skeletal muscle; AWGS, Asia Working Group for Sarcopenia; EWGSOP, European Working Group on Sarcopenia in Older People; SMI, skeletal muscle mass index; BIA, bioelectrical impedance analysis; DXA, Dual-energy X-ray absorptiometry; TUG, Timed up and go*.

The results for methodological quality assessment are summarized in Table 2. The study quality scores ranged from 4 to 8, of which 88% of studies were rated as good quality, 12% of studies were rated as fair quality, and all studies were RCTs. The process of concealed allocation was used in seven studies. The methods of single- or double-blinding of assessor and intention-to-treat analysis were used in nine studies (34, 36, 38–40, 42–44, 46) and seven studies (31, 32, 38, 39, 41, 44, 46), respectively. As for the other characteristics, such as similarity on key measures at baseline, comparison with more than one outcome was fully reported in the included studies.

**Table 2:** Methodological quality of the included studies [The Physiotherapy Evidence Database (PEDro analysis)].

**Study**	**Score**	**Methodological quality**	**PEDro item number**										
			**1**	**2**	**3**	**4**	**5**	**6**	**7**	**8**	**9**	**10**	**11**
Chen et al. 2018 (31)	6	Good	1	1	0	1	0	0	0	1	1	1	1
Hassan et al. 2016 (32)	6	Good	1	1	0	1	0	0	0	1	1	1	1
I Iranzo et al. 2018 (40)	6	Good	1	1	0	1	0	0	1	1	0	1	1
Jung et al. 2019 (41)	6	Good	1	1	0	1	0	0	0	1	1	1	1
Kim et al. 2012 (42)	7	Good	1	1	1	1	0	0	1	1	0	1	1
Kim et al. 2013 (43)	7	Good	1	1	1	1	0	0	1	1	0	1	1
Lichtenberg et al. 2019 (44)	7	Good	1	1	0	1	0	0	1	1	1	1	1
Mafi et al. 2019 (45)	6	Good	1	1	1	1	0	0	0	1	0	1	1
Makizako et al. 2020 (46)	7	Good	1	1	0	1	0	0	1	1	1	1	1
Maruya et al. 2016 (47)	5	Fair	1	1	0	1	0	0	0	1	0	1	1
Piastra et al. 2018 (33)	5	Fair	1	1	0	1	0	0	0	1	0	1	1
Strasser et al. 2018 (34)	6	Good	1	1	0	1	0	0	1	1	0	1	1
Tsekoura et al. 2018 (35)	6	Good	1	1	1	1	0	0	0	1	0	1	1
Vikberg et al. 2019 (36)	7	Good	1	1	1	1	0	0	1	1	0	1	1
Wei et al. 2016 (37)	6	Good	1	1	1	1	0	0	0	1	0	1	1
Yamada et al. 2019 (38)	7	Good	1	1	0	1	0	0	1	1	1	1	1
Zhu et al. 2019 (39)	8	Good	1	1	1	1	0	0	1	1	1	1	1

*Studies were classified as having excellent (9–10), good (6–8), fair (4–5), or poor (< 4).*

*Scale of item score: 0, absent; 1, present. The PEDro scale criteria are (1) eligibility criteria, (2) random allocation, (3) concealed allocation, (4) similarity at baseline on key measures, (5) subject blinding, (6) therapist blinding, (7) assessor blinding, (8) more than 85% follow-up of at least 1 key outcome, (9) intention-to-treat analysis, (10) between-group statistical comparison for at least one key outcome, and (11) point estimates and measures of variability provided for at least one key outcome*.

### Synthetic Results

**Figure 2:**
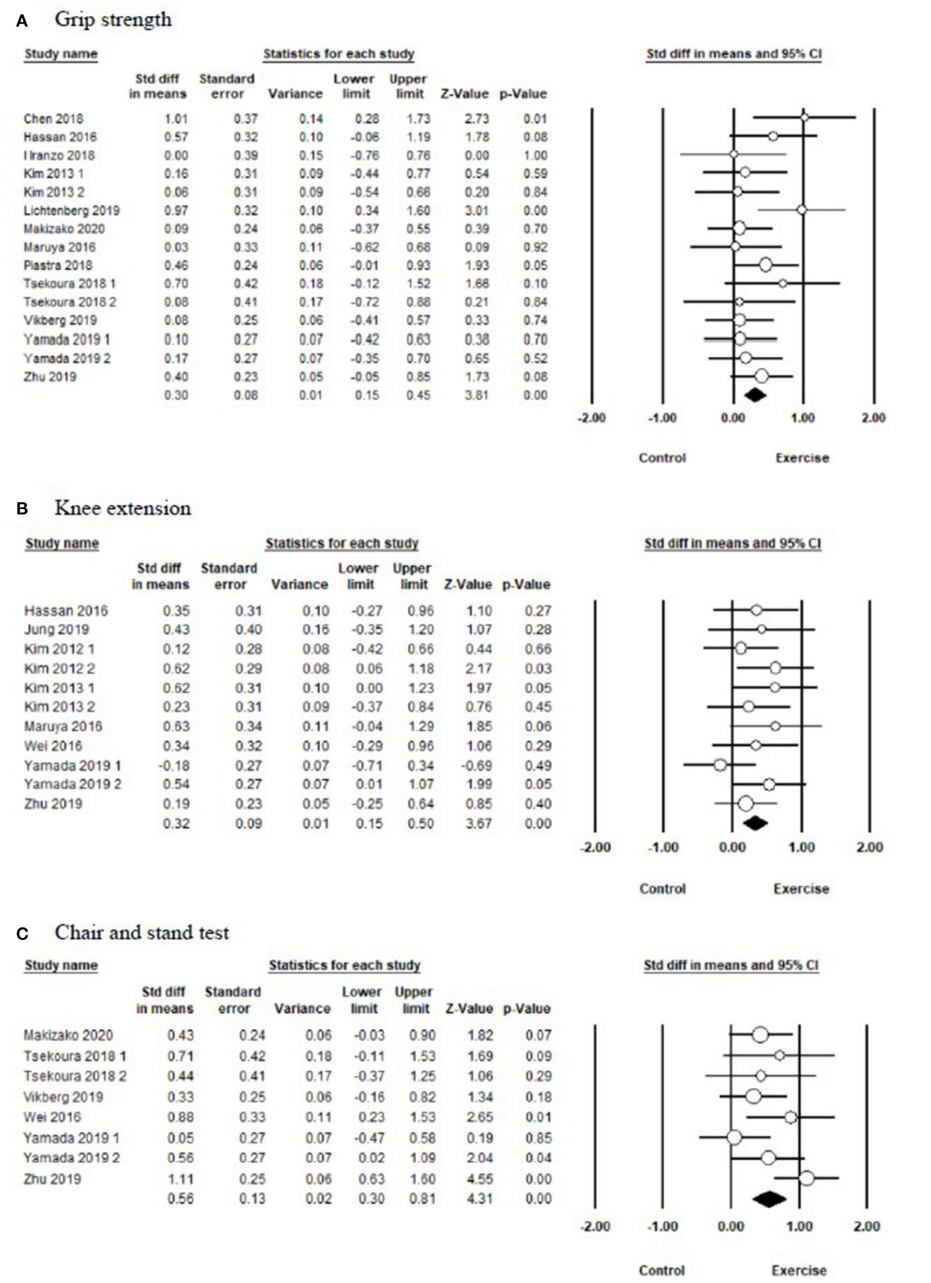
Forest plot showing the effects of exercise vs. control on muscle strength: **(A)** grip strength, **(B)** knee extension, **(C)** chair and stand test.

In terms of muscle strength (Figure 2 and Table 3), there were 12 studies, including 15 parallel comparisons on exercise and control conditions (as three studies included two paired trials, respectively), on measuring the grip strength. A pooled comparison revealed that exercise intervention had significant improvement in grip strength (SMD = 0.30, 95% CI [0.15, 0.45], *I*^2^ = 6%, *p* < 0.01). Pooled results from eight trials revealed a significant improvement in chair and stand [SMD = 0.56, 95% CI (0.30, 0.81), *I*^2^ = 36%, *p* < 0.01] compared with the control group. Moreover, pooled analysis from 11 parallel trials showed a significant improvement in knee extension in favor of exercise intervention [SMD = 0.32, 95% CI (0.15, 0.50), *I*^2^ = 0%, *p* < 0.01) compared with the control group.

**Table 3:** Synthesized results for the effects of exercise vs control intervention.

**Variables**	***k***	***SMD***	**95% CI**	***I*^2^%**	**Between-group homogeneity**			**Publication bias**
					***Q*-value**	**df(*Q*)**	***p*-value**	**Egger's test (*p*)**
**Muscle strength**								
Grip strength	15	0.30	0.15–0.45	6	14.90	14	0.39	0.39
Knee extension	11	0.32	0.15–0.50	0	7.99	10	0.63	0.19
Chair and stand	8	0.56	0.30–0.81	36	10.95	7	0.14	0.86
**Physical performance**								
TUG	9	0.74	0.48–1.00	31	11.63	8	0.17	0.01
Gait speed	17	0.59	0.35–0.82	62	41.74	16	0.001	0.01
**Body composition**								
Skeletal mass index	11	0.37	0.15–0.58	16	11.90	10	0.29	0.92
ASM	13	0.31	0.13–0.49	20	15.07	12	0.24	0.21
Lean mass	4	0.20	−0.07 to 0.48	0	0.80	3	0.85	0.20
Body fat	5	0.24	−0.04 to 0.53	3	4.12	4	0.39	0.15
Fat-free mass	3	0.36	−0.10 to 0.82	0	0.36	2	0.84	0.39

*ASM, appendicular skeletal muscle; SMD, standard mean difference; TUG, timed up and go; k, number of trials*.

**Figure 3:**
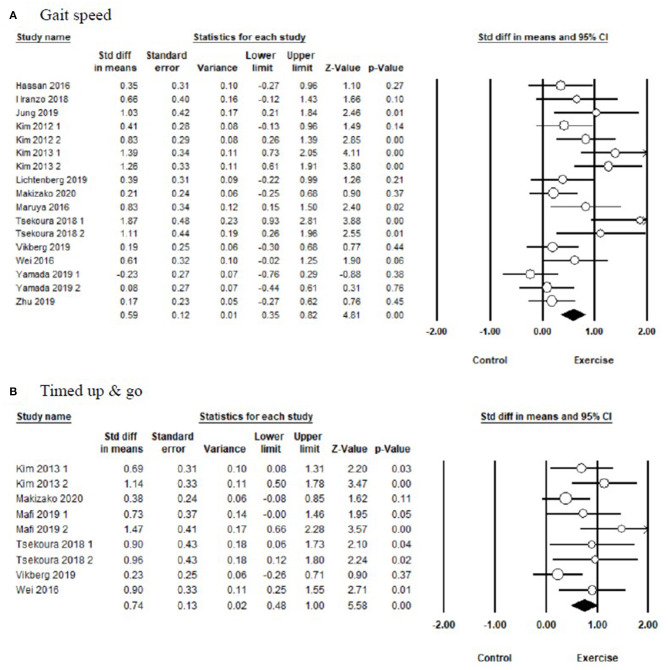
Forest plot showing the effects of exercise vs. control on physical performance: **(A)** gait speed, **(B)** timed up and go test.

In terms of physical performance (Figure 3 and Table 3), the pooled results showed that exercise produced significant improvements in TUG [SMD = 0.74, 95% CI (0.48, 1.00), *I*^2^ = 0%, *p* < 0.01], and gait speed [SMD = 0.59, 95% CI (0.35, 0.82), *I*^2^ = 62%, *p* < 0.01] compared with the control group.

**Figure 4:**
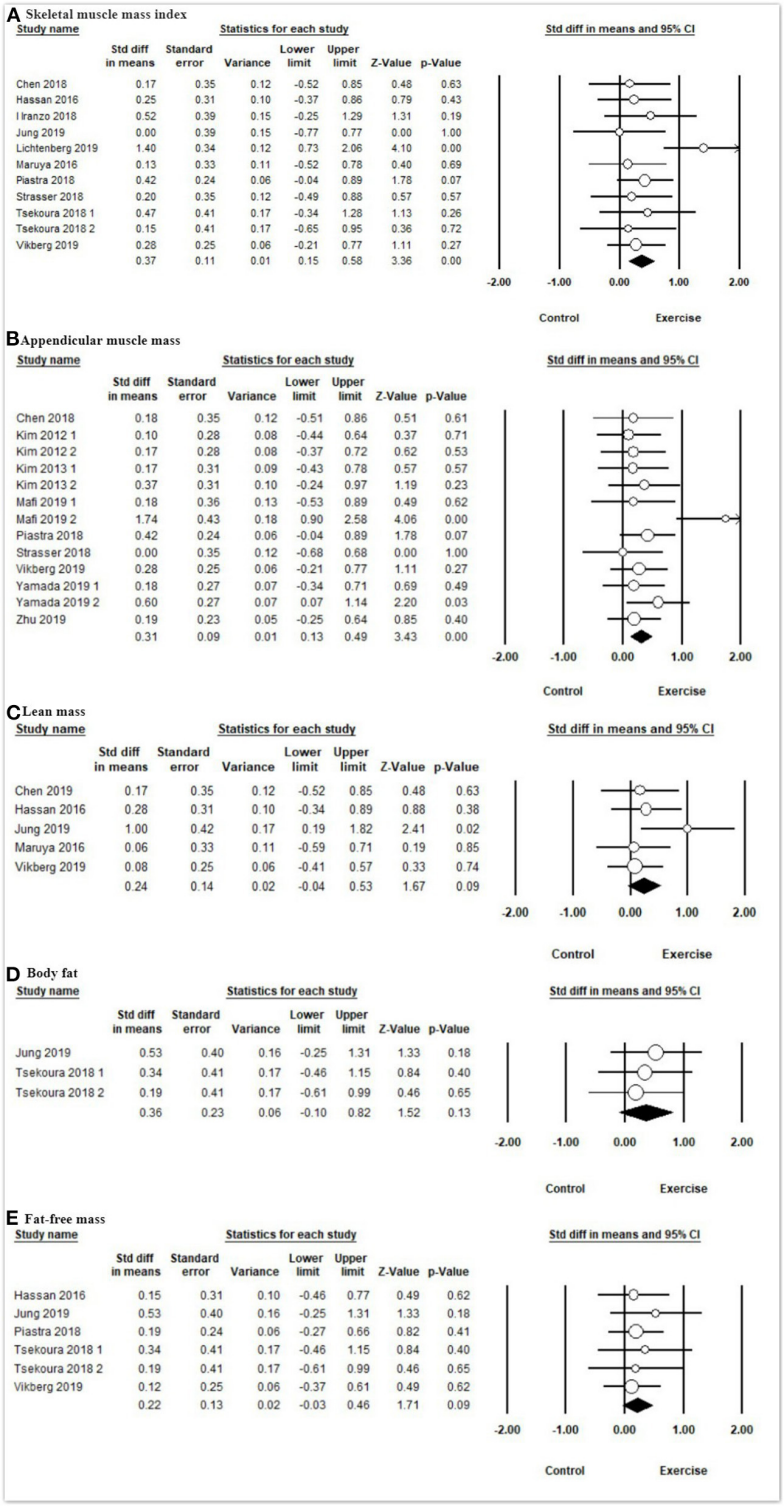
Forest plot showing the effects of exercise vs. control on body composition: **(A)** skeletal muscle mass index, **(B)** appendicular muscle mass, **(C)** lean mass, **(D)** body fat, **(E)** fat-free mass.

In terms of body composition (Figure 4 and Table 3), the meta-analysis presented that exercise had significant improvements in SMI [SMD = 0.37, 95% CI (0.15, 0.58), *I*^2^ = 16%, *p* < 0.01] and ASM [SMD = 0.31, 95% CI (0.13, 0.49), *I*^2^ = 20%, *p* < 0.01] compared with the control group, but there were no significant differences on lean mass [SMD = 0.20, 95% CI (−0.07, 0.48), *I*^2^ = 0%, *p* = 0.15], body fat [SMD = 0.24, 95% CI (−0.04, 0.53), *I*^2^ = 3%, *p* = 0.09], and fat-free mass [SMD = 0.36, 95% CI (−0.10, 0.82), *I*^2^ = 0%, *p* = 0.13] between the exercise group and control group.

### Moderator Analysis

To investigate the moderator effects of exercise on interested outcomes, moderator analyses were conducted according to the categorical and continuous variables in Table 4. The effects of exercise on TUG (*Q* = 4.45, *df* = 1, *p* = 0.04) and SMI (*Q* = 7.90, *df* = 2, *p* = 0.02) were significantly moderated by exercise intensity. Moderate–vigorous intensity [SMD = 0.81, 95% CI (0.57, 1.05), *p* < 0.01] of exercise significantly improved TUG compared with the high intensity [SMD = 0.23, 95% CI (−0.26, 0.71), *p* = 0.37] of exercise. The high intensity [SMD = 1.39, 95% CI (0.73, 2.06), *p* < 0.01] and moderate intensity [SMD = 0.41, 95% CI (0.10, 0.72), *p* < 0.01] of exercise significantly improved SMI compared with the light-to-moderate intensity [SMD = 0.29, 95% CI (−0.20, 0.79), *p* = 0.25] of exercise.

**Table 4:** Moderator analysis for the effects of exercise on measurement outcomes.

**Variables**	**Muscle Strength**			**Physical Performance**		**Body Composition**				
	**Grip Strength**	**Knee Extension**	**Chair and Stand**	**TUG**	**Gait Speed**	**ASM**	**SMI**	**Muscle Mass**	**Lean Mass**	**BODY FAT**
	**SMD (95% CI)**	**SMD (95% CI)**	**SMD (95% CI)**	**SMD (95% CI)**	**SMD (95% CI)**	**SMD (95% CI)**	**SMD (95% CI)**	**SMD (95% CI)**	**SMD (95% CI)**	**SMD (95% CI)**
**Criteria**										
AWGS	0.23 (0.03–0.43)	0.32 (0.13–0.51)	0.55 (0.11–0.98)	0.69 (0.26–1.13)	0.55 (0.23–0.88)	0.25 (0.05–0.44)	0.18 (−0.13 to 0.49)	0.08 (−0.21 to 0.36)	0.53 (−0.25 to 1.31)	0.36 (−0.18 to 0.91)
EWGSOP	0.41 (0.15–0.67)	0.34 (−0.10 to 0.78)	0.54 (0.22–0.87)	0.79 (0.43–1.16)	0.64 (0.28–1.00)	0.47 (0.00–0.94)	0.49 (0.18–0.80)	0.19 (−0.20 to 0.57)	0.16 (−0.14 to 0.45)	0.16 (−0.22 to 0.54)
**Sex**										
Female	0.39 (0.03–0.76)	0.39 (0.12–0.66)	–	0.91 (0.46–1.35)	0.95 (0.59–1.31)	0.25 (0.02–0.48)	0.27 (−0.07 to 0.62)	0.13 (−0.11 to 0.37	0.28 (−0.12 to 0.68)	0.55 (−0.26 to 1.37)
Male	0.97 (0.34–1.60)	–	–	1.08 (0.35–1.81)	0.39 (−0.22 to 0.99)	0.94(−0.59 to 2.48)	1.40 (0.73–2.06)^*^	–	–	–
Mixed	0.21 (0.03–0.39)	0.28 (0.05–0.51)	0.5 (0.30–0.81)	0.26 (0.26–0.86)	0.43 (0.15–0.70)	0.27 (0.03–0.50)	0.27 (0.02–0.52)	0.001 (−0.68 to 0.68)	0.13 (−0.25 to 0.52)	0.13 (−0.20 to 0.46)
**Exercise type**										
RT	0.33 (0.14–0.51)	0.32 (0.09–0.54)	0.31 (0.02–0.61)	0.80 (0.37–1.23)	0.50 (0.19–0.81)	0.33 (0.13–0.52)	0.54 (0.15–0.74)	0.11 (−0.11 to 0.34)	0.16 (−0.14 to 0.45)	0.16 (−0.17 to 0.49)
RT+AE	0.24 (−0.02 to 0.50)	0.35 (0.01–0.68)	0.71 (0.33–1.08)	0.59 (0.23–0.96)	0.92 (0.35–1.50)	0.19 (−0.25 to 0.64)	0.18 (−0.20 to 0.55)	–	0.53 (−0.25 to 1.31)	0.50 (−0.42 to 1.42)
WBV	–	0.34 (−0.29 to 0.96)	0.88 (0.23–1.53)	0.90 (0.25–1.55)	0.61 (−0.02 to 1.25)	–	–	–	–	–
**Exercise frequency**										
≥3 times/week	0.14 (−0.16 to 0.44)	0.46 (0.07–0.85)	0.72 (0.39–1.04)	0.79 (0.43–1.16)	0.85 (0.41–1.29)	0.68 (−0.18 to 1.55)	0.25 (−0.02 to 0.53)		0.24 (−0.18 to 0.65)	0.31 (−0.22 to 0.83)
<3 times/week	0.36 (0.16–0.55)	0.29 (0.10–0.48)	0.35 (0.06–0.64)	0.69 (0.26–1.13)	0.47 (0.19–0.85)	0.26 (0.08–0.43)	0.48 (0.06–0.90)	0.11 (−0.11 to 0.34)	0.18 (−0.19 to 0.55)	0.23 (−0.23 to 0.68)
**Exercise duration**										
>12 weeks	0.39 (0.12–0.65)	0.33 (0.02–0.65)	0.77 (0.11–1.44)	–	0.38 (0.02–0.75)	0.25 (−0.04 to 0.54)	0.28 (−0.01 to 0.58)	0.19 (−0.20 to 0.57)	0.18 (−0.19 to 0.55)	0.18 (−0.27 to 0.62)
≤ 12 weeks	0.27 (0.06–0.47)	0.32 (0.11–0.53)	0.44 (0.19–0.68)	0.74 (0.48–1.00)	0.63 (0.35–0.92)	0.34 (0.11–0.57)	0.43(0.08–0.79)	0.08 (−0.21 to 0.36)	0.24 (−0.18 to 0.65)	0.34 (−0.17 to 0.86)
**Session time**										
≤ 45min	0.14 (−0.09 to 0.37)	0.31 (−0.07 to 0.68)	0.44 (0.19–0.68)	0.66 (0.25–1.06)	0.55 (0.15–0.96)	0.35 (0.05–0.64)	0.29 (−0.01 to 0.59)		0.12 (−0.37 to 0.61)	0.07 (−0.31 to 0.46)
>45min	0.41 (0.19–0.65)	0.34 (0.12–0.56)	0.77 (0.11–1.44)	0.82 (0.44–1.19)	0.62 (0.33–0.92)	0.30 (0.07–0.53)	0.42 (0.04–0.79)	0.11 (−0.11 to 0.34)	0.24 (−0.09 to 0.58)	0.43 (−0.04 to 0.90)
**Exercise intensity**										
Light	0.22 (−0.01 to 0.45	0.27 (−0.08 to 0.61)	0.58 (0.03–1.20)	–	0.24 (−0.11 to 0.59)	0.31 (0.02–0.60)	0.29 (−0.20 to 0.79)	–	–	0.06 (−0.59 to 0.71)
Moderate-vigorous	0.35 (0.09–0.61)	0.38 (0.13–0.62)	0.58 (0.26–0.90)	0.81 (0.57–1.05)	0.82 (0.48–1.16)^*^	0.32 (0.06–0.59)	0.30 (0.03–0.56)^*^	0.11 (−0.11 to 0.34)	0.18 (−0.189 to 0.552)	0.16 (−0.22 to 0.54)
Vigorous	0.50 (−0.37 to 1.37)	0.43 (−0.35 to 1.20)	0.33 (−0.16 to 0.82)	0.23 (−0.26 to 0.71)	0.44 (0.01 to 0.87)	0.28 (−0.21 to 0.77)	0.56 (−0.23 to 1.35)	–	0.24 (−0.18 to 0.65)	0.55 (−0.26 to 1.37)
**Control group**										
Active	0.30 (0.08–0.53)	0.28 (0.01–0.54)	0.31 (0.001–0.61)	0.70 (0.41–1.00)	0.76 (0.38–1.15)	0.23 (0.04–0.41)	0.48 (0.13–0.83)	0.14 (−0.14 to 0.42)	0.22 (−0.09 to 0.53)	0.48 (−0.41 to 1.38)
Passive	0.30 (0.07–0.53)	0.38 (0.13–0.63)	0.74 (0.41–1.06)	0.85 (0.24–1.46)	0.34 (0.12–0.56)	0.61 (0.03–1.20)	0.21 (−0.10 to 0.52)	–	0.15 (−0.46 to 0.77)	0.17 (−0.20 to 0.55)

* *p < 0.05.* *AE, aerobic exercise; ASM, appendicular skeletal muscle; AWGS, Asia Working Group for Sarcopenia; EWGSOP, European Working Group on Sarcopenia in Older People; RT, resistance training; TUG, timed up and go; WBV, whole-body vibration training*.

**Figure 5:**
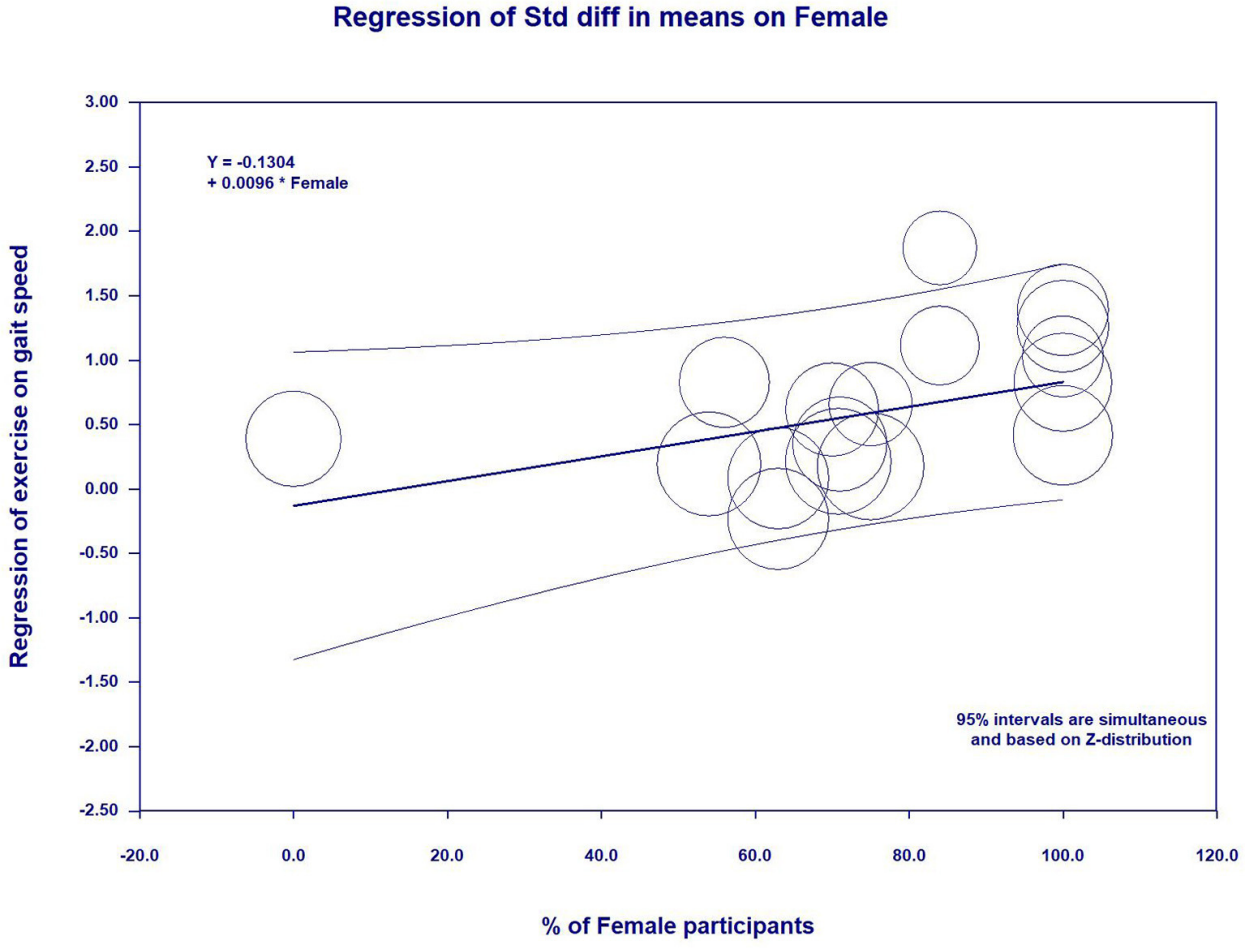
Effect sizes by percent of female participants in meta-regression for gait speed.

**Figure 6:**
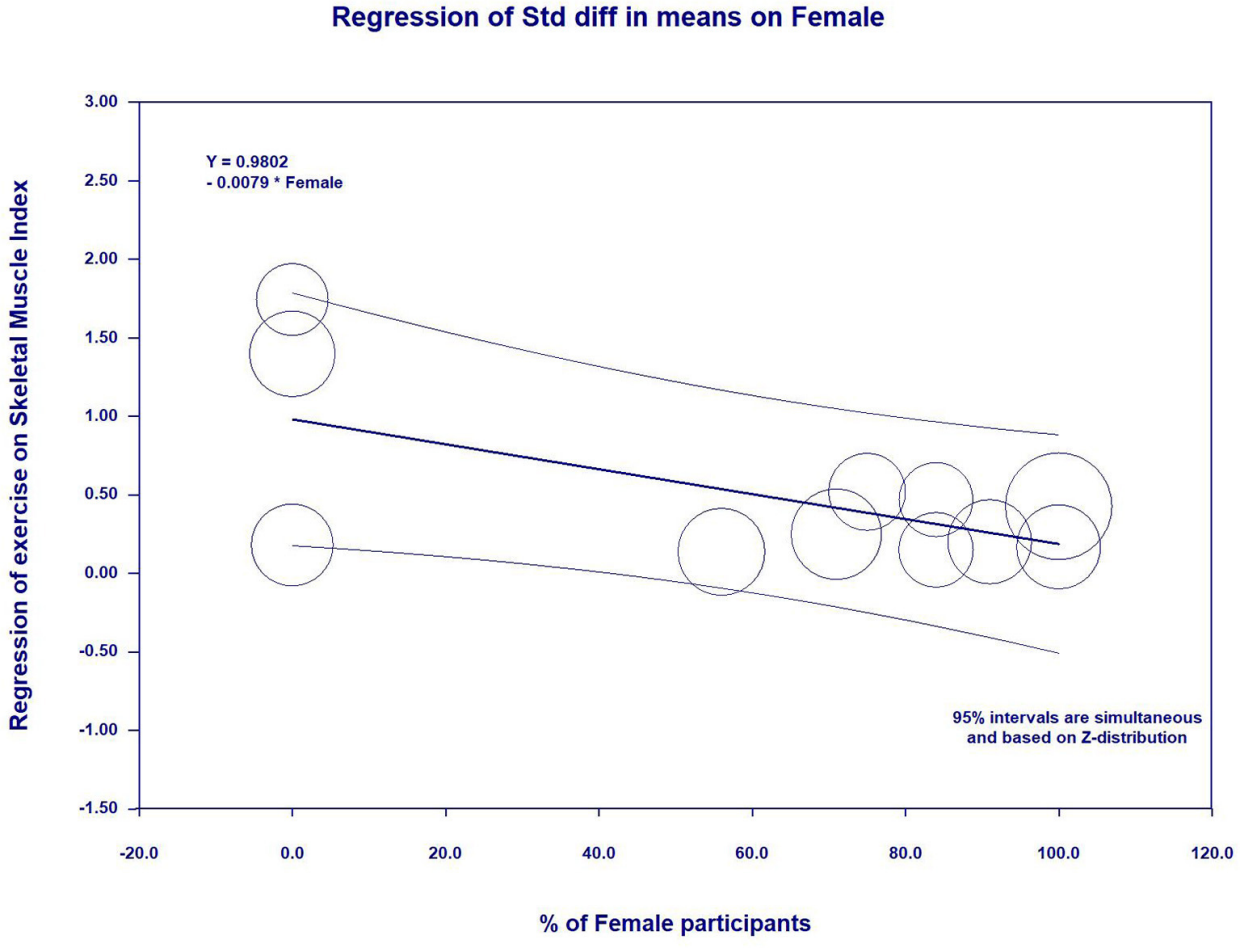
Effect sizes by percent of female participants in meta-regression for skeletal muscle index.

In the meta-regression, the percent of female participants in the original studies was significantly associated with the gait speed (β = 0.0096, 95%CI: 0.0006 to 0.0186, *p* = 0.03) (Figure 5 and Table 5) and skeletal muscle index (β = −0.0092, 95%CI: −0.0162 to −0.0021, *p* = 0.01) (Figure 6 and Table 5). In addition, there were no significant moderator effects in age, percent of female participants, exercise times per week, exercise duration, and dose of exercise intervention. Results of meta-regression are shown in Table 5.

**Table 5:** Meta-regression for continuous variables to predict exercise effects on measurement outcomes.

**Variables**	**Muscle strength**			**Physical performance**		**Body composition**				
	**Grip strength β(95% CI)**	**Knee extension β(95% CI)**	**Chair stand test β(95% CI)**	**TUG β(95% CI)**	**Gait speed β(95% CI)**	**ASM β(95% CI)**	**SMI β(95% CI)**	**Muscle mass β(95% CI)**	**Lean mass β(95% CI)**	**Body fat β(95% CI)**
Age	−0.0089	−0.0110	−0.0255	0.0069	−0.0252	−0.0134	0.0117	−0.0210	0.0007	0.0023
	(−0.0364 to 0.0186)	(−0.0474 to 0.0254)	(−0.0765 to 0.0255)	(−0.0618 to 0.0759)	(−0.0758 to 0.0254)	(−0.0443 to 0.0174)	(−0.0249 to 0.0483)	(−0.0726 to 0.0306)	(−0.0438 to 0.0452)	(−0.0100 to 0.0146)
Percent of female participants	−0.0021 (−0.0087 to 0.0045)	0.0025 (−0.0078 to 0.0128)	0.0018 (−0.0072 to 0.0438)	−0.0012 (−0.0094 to 0.0071)	0.0096 (0.0006 to 0.0186) ^*^	−0.0051 (−0.0107 to 0.0004)	−0.0092 (−0.0162 to −0.0021) ^*^	0.0144 (−0.0661 to 0.0949)	0.0036 (−0.0098 to 0.0170)	0.0096 (−0.0048 to 0.0240)
Exercise time per week in minutes	0.0021 (−0.0014 to 0.0056)	0.0021 (−0.0018 to 0.0060)	−0.0004 (−0.0083 to 0.0075)	0.0016 (−0.0041 to 0.0072)	0.0046 (−0.0009 to 0.0101)	0.0024 (−0.0033 to 0.0082)	−0.0055 (−0.0124 to 0.0014)	—	−0.0011 (−0.0398 to 0.0179)	−0.0010 (−0.0110 to 0.0089)
Exercise duration	0.0063	0.0011	−0.0141	−0.0292	−0.0172	−0.0037	−0.031	0.0073	−0.0012	−0.0049
	(−0.0129 to 0.0255)	(−0.0305 to 0.0328)	(−0.0657 to 0.0378)	(−0.2068 to 0.1485)	(−0.0705 to 0.0362)	(−0.0255 to 0.0182)	(−0.0275 to 0.0213)	(−0.0150 to 0.0297)	(−0.0255 to 0.0231)	(−0.0522 to 0.0425)
Dose of exercise intervention	0.00000 (−0.0001 to 0.0001)	0.0001 (−0.0001 to 0.0002)	0.0001 (−0.0002 to 0.0003)	0.0004 (−0.0005 to 0.0014)	0.0001 (−0.0002 to 0.0003)	−0.0001 (−0.0002 to 0.0002)	−0.0001 (−0.0002 to 0.0001)	0.0001 (−0.0001 to 0.0002)	−0.00001 (−0.0002 to 0.0002)	−0.00001 (−0.0003 to 0.0002)

* *p < 0.05.* *ASM, appendicular skeletal muscle; SMI, skeletal muscle mass index; TUG, timed up and go; –, the continuous variable in each study is equivalent*.

### Publication Bias

Publication bias was evaluated using Egger's test (in Table 3) and Funnel plot (in Supplementary Figures). Of which, although the asymmetrical Funnel plot and Egger's test (Egger's regression intercept = 6.13, *p* < 0.05), the Duval and Tweedie's trim and fill showed that five studies were missing on the left side of the mean effect. The adjusted value was SMD = 0.35, 95% CI (0.08, 0.61), which was substantially lower than our estimation (SMD = 0.59). There seems to be evidence for publication bias in this meta-analysis since studies with smaller effect sizes are not provided.

## Discussion

The present systematic review and meta-analysis consisting of 17 RCTs (985 individual participants, 726 female, with sarcopenia) investigated the effects of exercises on muscle strength, physical performance, and body composition. The main findings of this systematic review with meta-analysis showed that exercises had significant benefits on muscle strength (grip strength, knee extension, and chair-stand), physical performance (timed up and go, and gait speed), and body composition (skeletal muscle mass index and appendicular skeletal muscle) compared with the control group in older adults with sarcopenia. The effects of exercise on gait speed and SMI were moderated by sex in the study and exercise intensity. These results may be important for implementing exercise interventions for sarcopenic older adults in clinic.

Since the differences in diagnostic criteria for sarcopenia, according to different organizations, the EWGSOP updated the definition and diagnostic criteria in 2008. This definition highlights that muscle strength is a principal element in sarcopenia diagnosis and is a useful predictor of adverse outcomes in people with sarcopenia (2). The EWGSOP and AWGS recommend that handgrip strength and chair-stand test are suitable measures of muscle strength. In our study, muscle strength was measured using the handgrip strength, chair stand test, and knee extension strength. The findings from this meta-analysis demonstrated that after exercise, older adults with sarcopenia demonstrated significant improvements in muscle strength. More specifically, exercises had small effects for grip strength (15 trials, ES = 0.31) and knee extension strength (11 trails, ES = 0.36) and moderate effect for chair and stand (eight trials, ES = 0.56) compared with the control groups, respectively. Consistent with previous meta-analysis and systematic reviews (49, 50), exercise has significant effects on muscle strength in older adults. Increased muscle strength may be associated with the neuronal adaptations, such as increases in muscle fiber tissue or synchronization of muscle contractions (51). During exercise training, muscle fiber are re-structured, leading to an increase in neuronal activity that stimulates an increase in muscle strength (52).

In regard to physical performance, the common testing tools include gait speed and TUG. Previous studies have demonstrated that poor physical performance, similar to low muscle strength, is associated with a higher risk of death in older adults (53, 54). The latest EWGSOP consensus recommends using physical performance to assess the severity of sarcopenia. Herein, gait speed and TUG were used to measure the physical performance in this review study. Our meta-analysis found that exercise's benefit is improving gait speed and TUG, and the positive effects can be supported by a previous review study targeting older adults (55). These meaningful results are of great importance because there is a close relationship between muscle strength and physical performance (56). Consistent with another similar study, the study of Capodaglio et al. on older adults over 75 years found that significant improvements in walking ability and TUG were attributed to the improved lower limb strength after exercise training (57). Thus, our findings reinforce the important role of exercise in physical performance in older adults with sarcopenia.

With reference to the body composition, in particular, the use of DXA or BIA to measure skeletal muscle mass and appendicular skeletal muscle mass has been considered as an important approach to assess the muscle quantity or quality to identify sarcopenia in the latest consensus (2). On this basis, our meta-analysis study indicated that the SMI and ASM have been significantly improved after exercise in sarcopenic older adults, which was inconsistent with previous systematic reviews (22, 58). The possible reason is that the improved muscle mass indicates an anabolic potential of exercise, inducing muscle hypertrophy via resistance exercise or aerobic exercise such as cycling and walking among all of ages (59). More importantly, improved skeletal muscle mass might be attributed to the increase in size of slow muscle fibers (60) and the increase in fast-twitch fiber sizes (61). Moreover, the statistical power in our study was augmented through including more eligible studies than previous meta-analysis studies (22, 58). Thus, it is believed that older adults with sarcopenic can improve their muscle mass through appropriate exercise.

In addition, the moderator analyses revealed that moderate to vigorous intensity exercise (ES = 0.81) could produce greater effects on TUG than did vigorous intensity exercise (ES = 0.23). This result may be inconclusive due to the small number of included studies investigating vigorous intensity exercise and the different methods on coding intensity exercise in the subgroup. Actually, a recent systematic review study has documented that both moderate and vigorous intensity exercises can improve functional ability in frail older adults (55). Future research is warranted to determine the effect of exercise at different intensities on physical performance. As for SMI, after practicing exercise at moderate and vigorous intensity, sarcopenic adults had significant improvement in their SMI compared with those practiced exercise at low intensity. This finding is similar to a previous study by Csapo et al., in which they found that high-intensity exercise had more advantage on increasing skeletal muscle mass than low-intensity exercise in older adults (19). Indeed, moderate to high-intensity exercise enhances the skeletal muscle mass via stimulating protein synthesis (62). On the contrary, lack of stimulation of the protein synthesis in muscle is related to low-intensity exercise, and it is recommended to increase skeletal muscle mass by compensating for more repetitions and velocity of motion (63). Despite this, it is recommended to explore the underlying mechanism of effects of exercise at different intensities

Additionally, the meta-regression revealed gender-specific effects on exercise-related changing in gait speed and SMI, implying a tendency that female participants had more improvements in gait speed and SMI than male participants after practicing exercise. The explanations for this findings may be attributed to external confounding (e.g., completed quality and motivation of the participants) that might affect the results (36). Based on this hypothesis, gender-specific effects did contribute to significant differences on other outcomes, such as grip strength, chair and stand, TUG, etc. Further studies are warranted to investigate the gender difference on the effects of exercise on physical performance and skeletal muscle mass.

Our systematic review and meta-analysis have some strengths that should be noted. All studies included in this meta-analysis were RCTs, which provided the empirical data for understanding the evidence of a treatment's efficacy. Furthermore, participants in this present study were only sarcopenic older adults without other physical conditions, like not being obese; so our research findings can be applied to the prevention or treatment in this sarcopenia population. Additionally, other potential confounders were examined to find whether they had any influence on the effects of exercise. This novelty could provide more information for future research to look at the influences of these confounders.

There are, however, several limitations in our study. First, as there were no consistent assessment criteria for sarcopenia, participants who met the initial sarcopenia defined by the EWGSOP and AWGS were included in our study. It may result in publication bias. Herein, according to the latest operational definition of sarcopenia (e.g., EWGSOP, AWGS), uniform cutoff points are expected to diagnose the subject and measure the outcomes in the future study. Second, the included studies used different instruments to measure the interested outcomes such as body composition (e.g., BIA, DEXA), which will contribute to the effect size of outcomes. While both of them used to examine the sarcopenia in clinical research, which is suggested by EWGSOP and AWGS, the multifrequency BIA equivalent to the DEXA measurements could be used in future research so as to ensure the accuracy of the diagnosis. Moreover, confirming the effect of exercise on interested outcomes by analyzing different instruments used to measure muscle mass separately is warranted. Third, studies using a sole exercise intervention to treat sarcopenia were included, excluding studies combing exercise intervention and nutrition. As a source of muscle synthesis, nutrient intake methods are important for the prevention and treatment of sarcopenia. There is a need for further research that examines the effect of combined exercise and nutrition intervention on sarcopenia. Four, the percentage of the female participants (female 74%) was considerably high compared with male participants, and the findings may not generalize all populations. As a result, further study may benefit from investigating the effects of exercise on sarcopenia in males and females separately due to physical difference in gender.

## Conclusion

These meta-analysis results suggest that exercise interventions have positive effects on muscle strength, physical performance, and skeletal muscle mass for sarcopenic elderly, but no effect is found in body composition (e.g., fat mass, lean mass, and fat-free mass). Further researches need to use the latest consensus criteria proposed by EWGSOP or AWGS to identify the sarcopenia, and a larger number of studies are recommended to confirm our findings. Meanwhile, the effective exercise protocol should be designed as promoting strategies in treating sarcopenia.

## Data Availability Statement

The original contributions generated for the study are included in the article/Supplementary Material, further inquiries can be directed to the corresponding author.

## Author Contributions

YZ and WS conceptualized the study. YZ, LZ, JB, and XL handled the methodology. YZ, S-TC, DK, and XL were in charge of the software. DK, XL, and WS did the validation. YZ, LZ, and S-TC performed the formal analysis. YZ, LZ, S-TC, and JB conducted the investigation. YZ and XL were in charge of the resources. YZ, JB, and XL did the data curation. YZ, LZ, and XL prepared and wrote the original draft. LZ and WS reviewed, edited, and wrote the manuscript. YZ, S-TC, and DK did the visualization. WS was in charge of the supervision and project administration. All authors have read and agreed to the published version of the manuscript.

## Conflict of Interest

The authors declare that the research was conducted in the absence of any commercial or financial relationships that could be construed as a potential conflict of interest.
