# Supplementary material for: Effects and Moderators of Exercise on Sarcopenic Components in Sarcopenic Elderly: A Systematic Review and Meta-Analysis
Source: Front Med (Lausanne). 2021 May 19;8:649748. doi: 10.3389/fmed.2021.649748 (PMC8169963; doi:10.3389/fmed.2021.649748)
Supplement: Supplementary file 1 [file Data_Sheet_1.docx]

Supplementary Material

# Supplementary Figures

**Supplementary Figure 1.** The figure legends are required to have the same font as the main text, 12 point normal Times New Roman, single spaced. Please use a single paragraph for each legend and prepare the figures keeping in mind the PDF layout.

**Supplementary Appendix – Electronic searches**

- Medline and Embase

#1 "physical activity" [Title/Abstract]

#2 "physical therapy" [Title/Abstract]

#3 "resistance training" [Title/Abstract]

#4 **“aerobic exercise”** [Title/Abstract]

#5 "exercis*" [Title/Abstract]

#6 "train*"[Title/Abstract]

#7 #1 OR #2 OR #3 OR #4 OR #5 OR #6

#8 "sarcopenia" [Title/Abstract]

#9 "muscular atrophy" [Title/Abstract]

#10 "muscular weight" [Title/Abstract]

#11"grip strength" [Title/Abstract]

#12 "physical function" [Title/Abstract]

#13 #8 OR #9 OR #10 OR #11 OR #12

#14 "randomized controlled trials"[Title/Abstract]

#15 "clinical trial" [Title/Abstract]

#16 "random allocation" [Title/Abstract]

#17 #14 OR #16 OR #16

#18 "older adults" [Title/Abstract]

#19 "aged" [Title/Abstract]

#20 "elder*" [Title/Abstract]

#21 #18 OR #19 OR #20

#22 #7 AND #13 AND #21

- Cochrane library

#1 exercis*:ti,ab,kw, JOR “training”: ti,ab,kw, OR “physical activity”:ti,ab,kw, OR physical therapy: ti,ab,kw, OR walk*: ti,ab,kw OR aerobic program:ti,ab,kw, OR strength program: ti,ab,kw, OR resistance program: ti,ab,kw

#2 sarcopenia:ti,ab,kw, or “muscular mass”: ti,ab,kw, “muscle atrophy”:ti,ab,kw

#3 older adult*:ti,ab,kw OR aged:ti,ab,kw OR elder*:ti,ab,kw

#4 randomized controlled trial:ti,ab,kw OR random allocation :ti,ab,kw OR controlled trial:ti,ab,kw

#5 #1 AND #2 AND #3 AND #4

- SportDiscus and CLNAHL

#1 exercis*:ti,ab,kw, JOR “training”: ti,ab,kw, OR “physical activity”:ti,ab,kw, OR physical therapy: ti,ab,kw, OR walk*: ti,ab,kw OR aerobic program:ti,ab,kw, OR strength program: ti,ab,kw, OR resistance program: ti,ab,kw

#2 sarcopenia:ti,ab,kw, or “muscular mass”: ti,ab,kw, “muscle atrophy”:ti,ab,kw

#3 older adult*:ti,ab,kw OR aged:ti,ab,kw OR elder*:ti,ab,kw

#4 randomized controlled trial:ti,ab,kw OR random allocation :ti,ab,kw OR controlled trial:ti,ab,kw

#5 #1 AND #2 AND #3 AND #4

- SCOPUS

#1 TITLE-ABS-KEY ("physical activity"  OR  "physical therapy"  OR  "aerobic program"  OR  "strength program"  OR  "exercis*"  OR  "train*"  OR  "resistance training"  OR  "aerobic training")

#2 TITLE-ABS-KEY ("sarcopenia"  OR  "muscular atrophy"  OR  "muscular weight" OR "muscle mass")

#3 TITLE-ABS-KEY ("older adults"  OR  "aged"  OR  "elder*")

#4 TITLE-ABS-KEY ("randomized controlled trials"  OR  "clinical trial" OR "random allocation")

#5 #1 AND #2 AND #3 AND #4


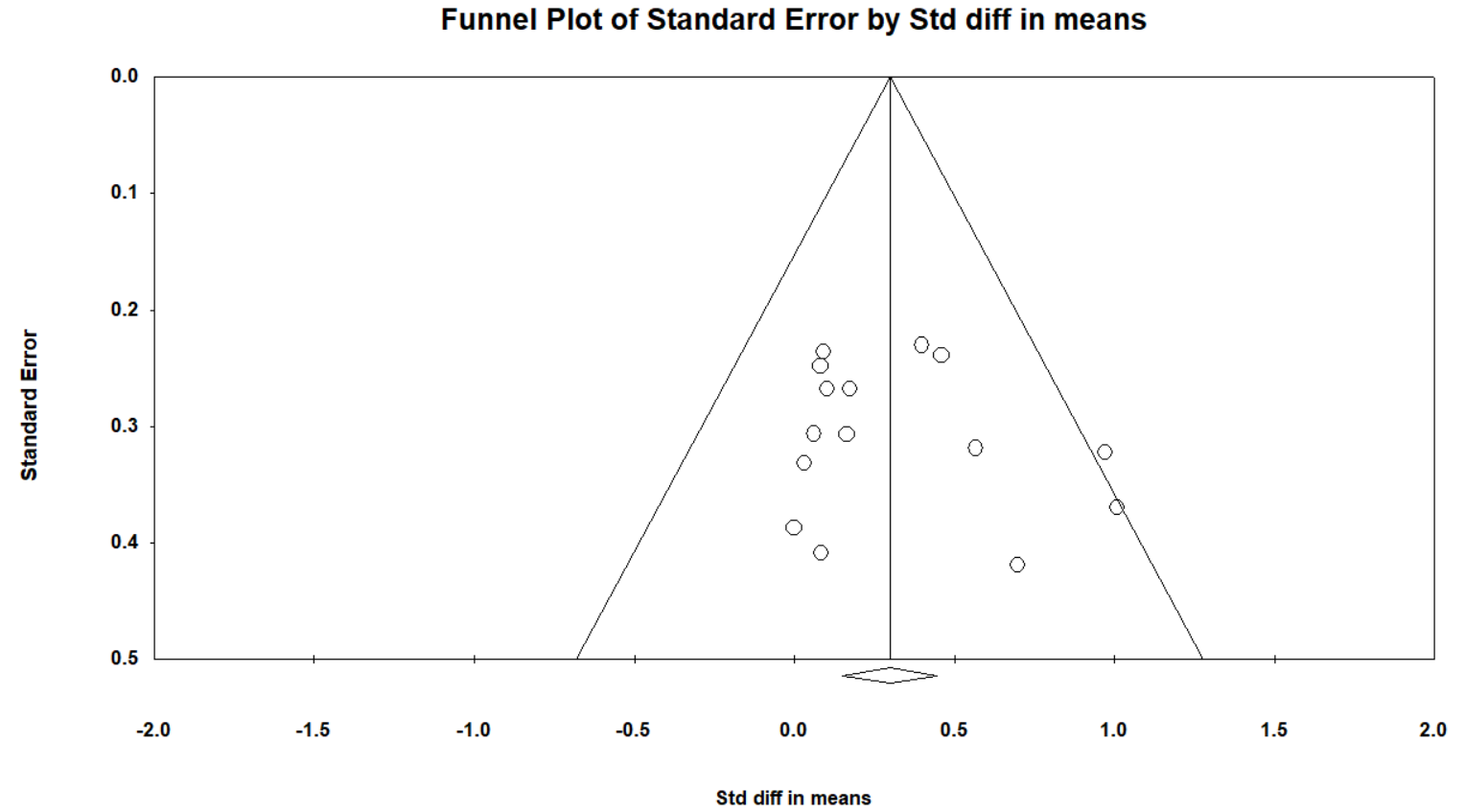


**Supplementary Figure 1**. Funnel plot estimating publication bias for Grip strength. Visual inspection indicates symmetrical distribution, and the Begg’s test was not statistically significant, suggesting an absence of publication bias (p=0.20).


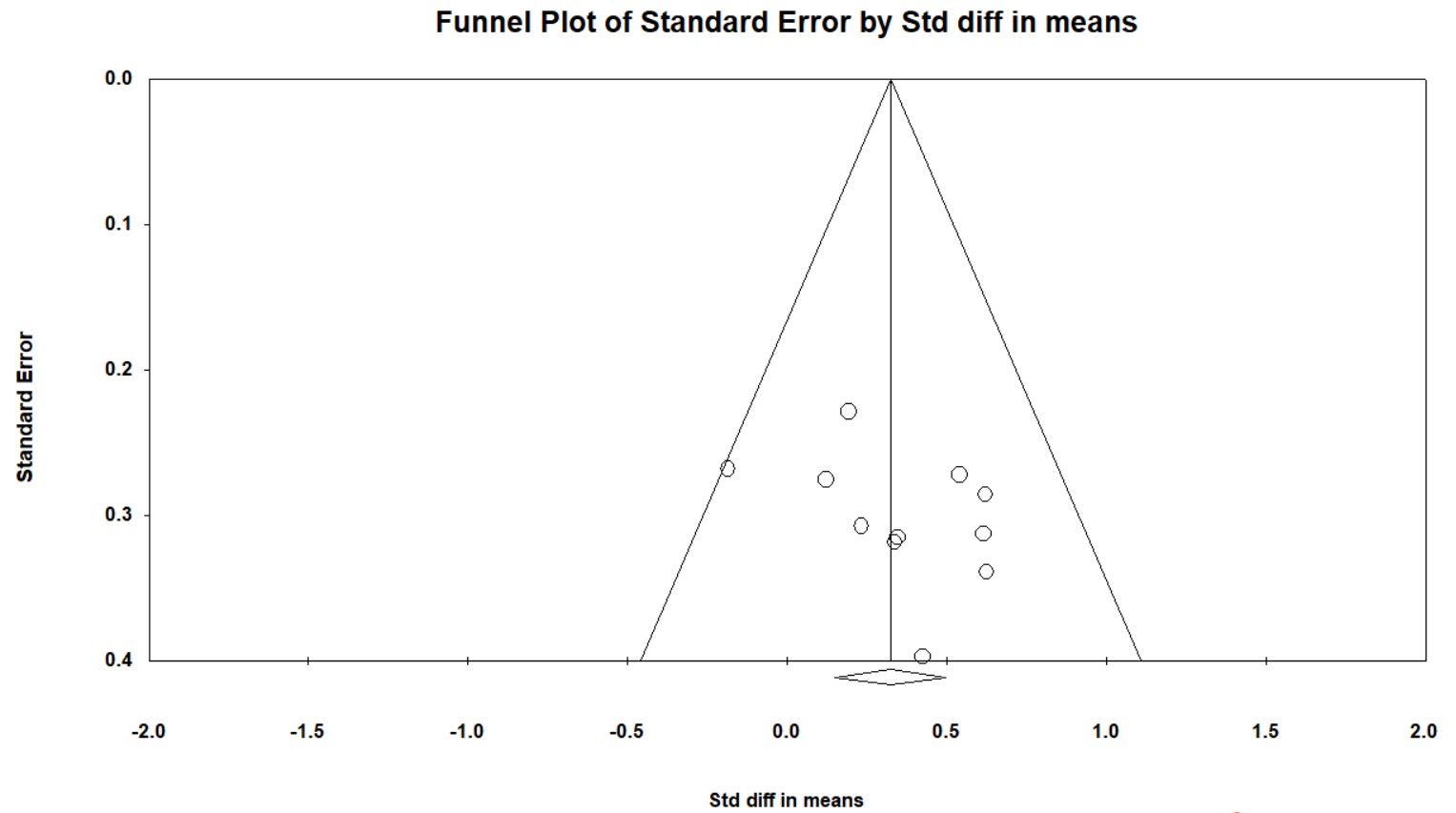


**Supplementary Figure 2**. Funnel plot estimating publication bias for Knee extension strength. Visual inspection indicates symmetrical distribution, and the Begg’s test was not statistically significant, suggesting an absence of publication bias (p=0.21).


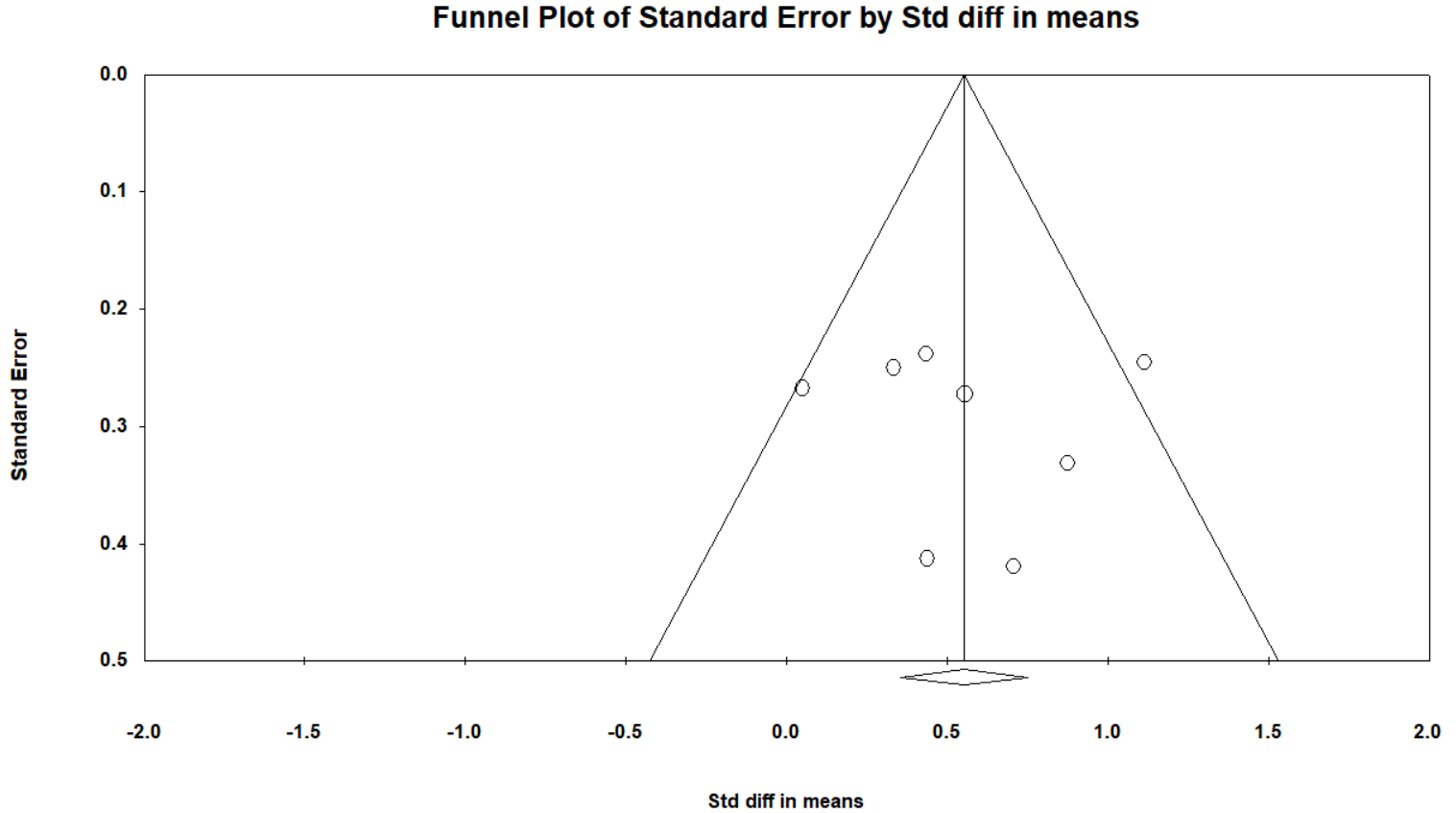


**Supplementary Figure 3.** Funnel plot estimating publication bias for Chair and stand test. Visual inspection indicates symmetrical distribution, and the Begg’s test was not statistically significant, suggesting an absence of publication bias (p=0.71).


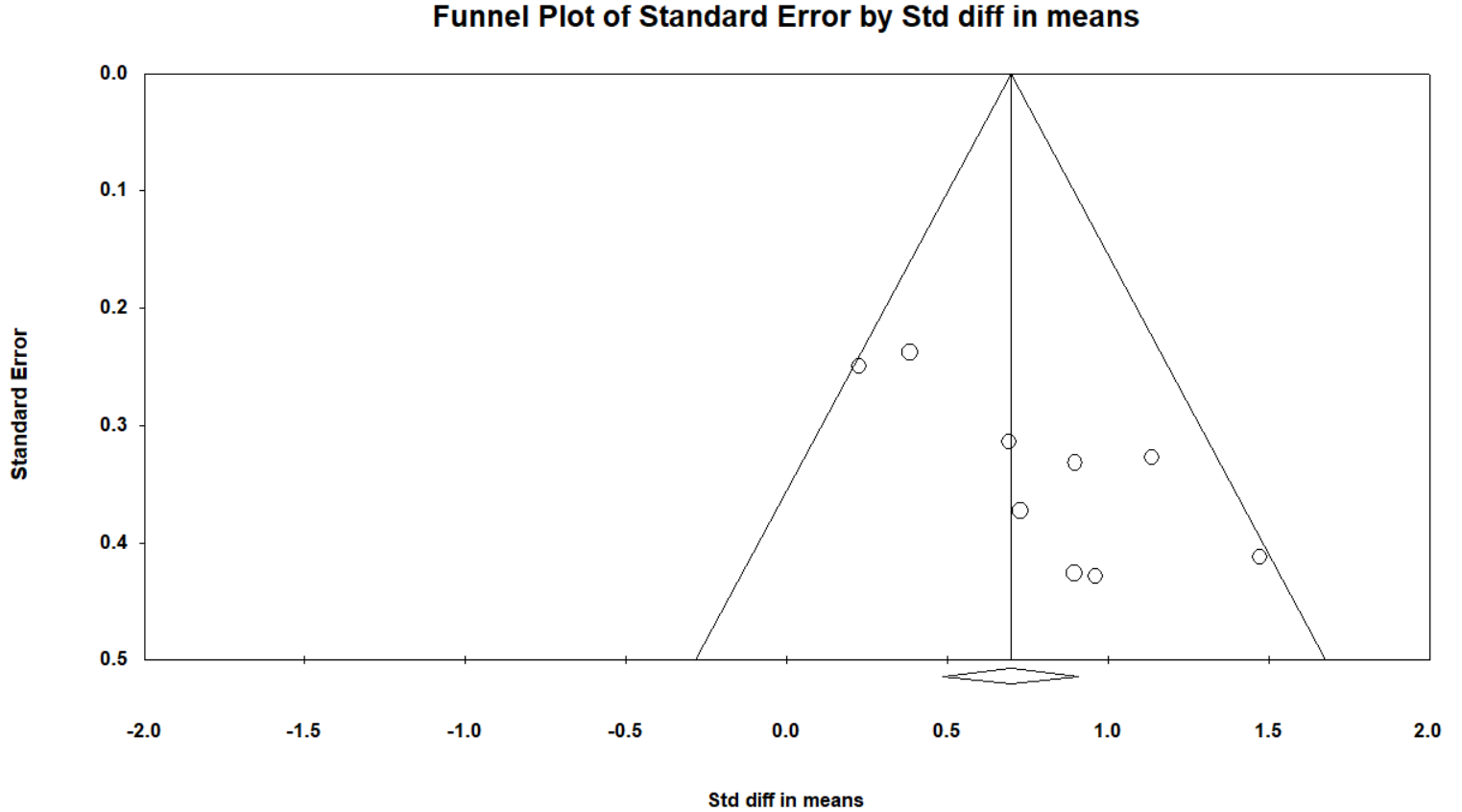


**Supplementary Figure 4.** Funnel plot estimating publication bias for Timed up and go. Visual inspection indicates symmetrical distribution, and the Begg’s test was not statistically significant, suggesting an absence of publication bias (p=0.12).


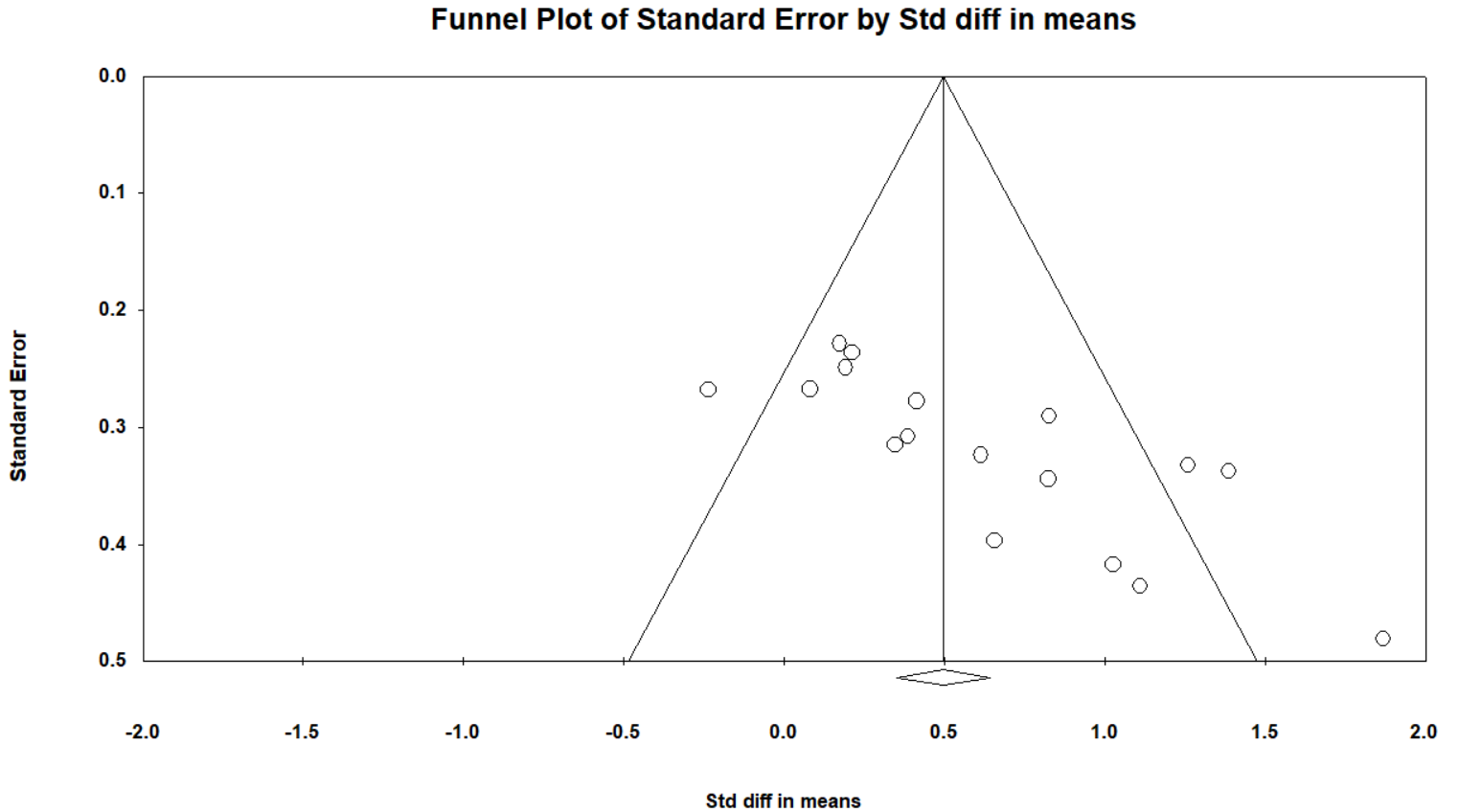


**Supplementary Figure 5.** Funnel plot estimating publication bias for Gait speed. Visual inspection indicates unsymmetrical distribution, and the Begg’s test was not statistically significant, suggesting a presence of publication bias (p<0.05).


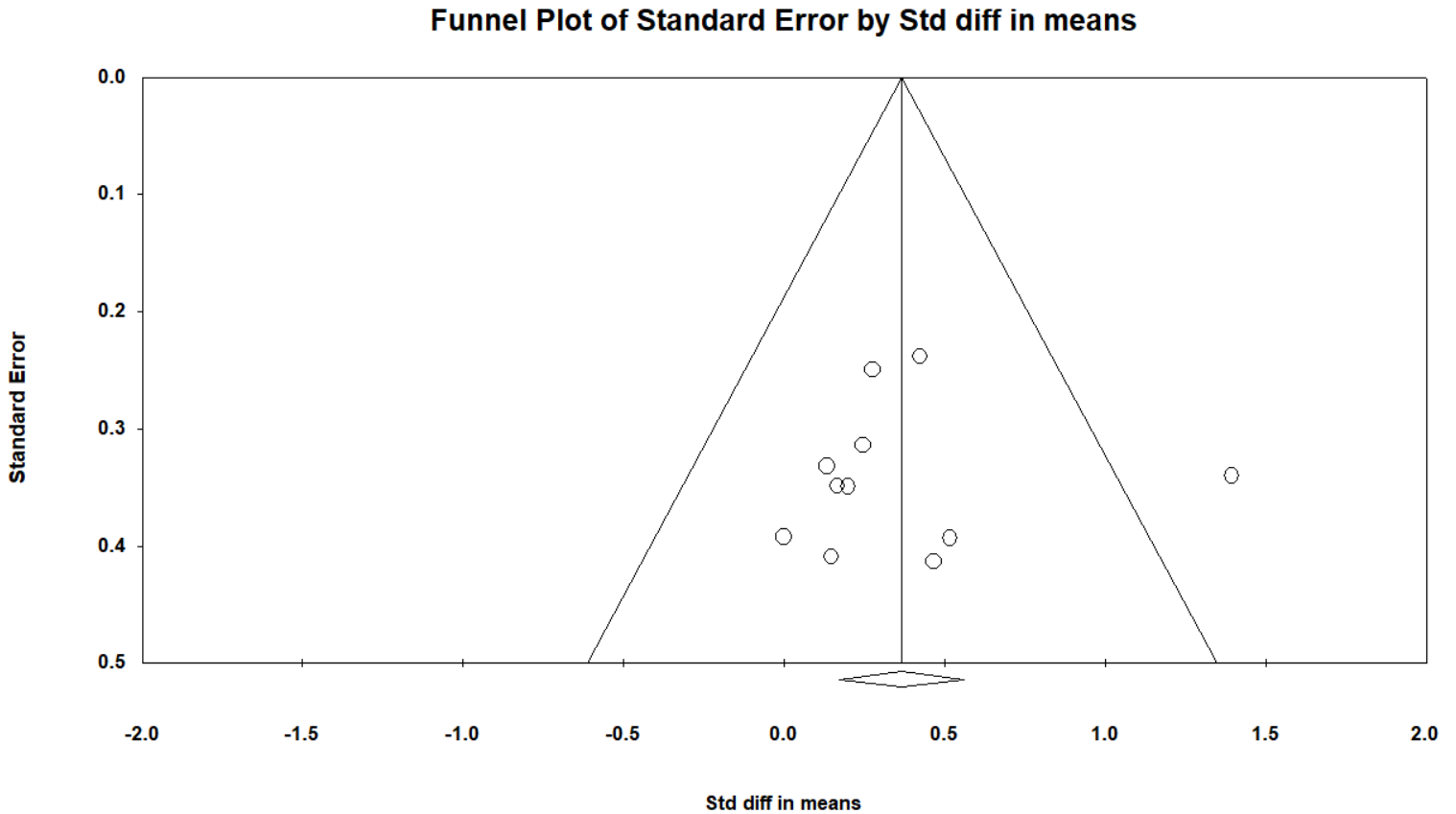


**Supplementary Figure 6.** Funnel plot estimating publication bias for Skeletal muscle index. Visual inspection indicates unsymmetrical distribution, and the Begg’s test was not statistically significant, suggesting an absence of publication bias (p=0.64).


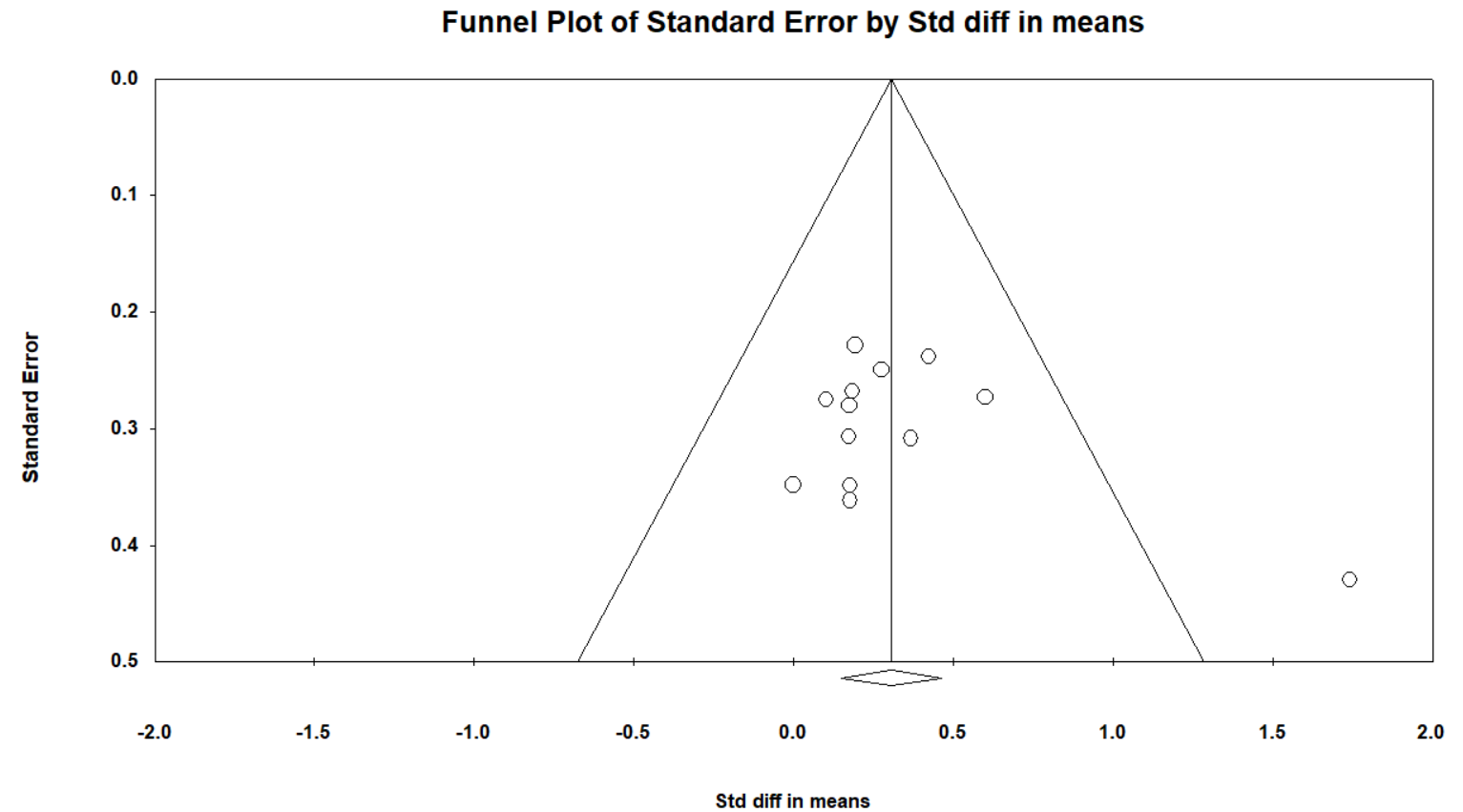


**Supplementary Figure 7**. Funnel plot estimating publication bias for appendicular muscle mass. Visual inspection indicates unsymmetrical distribution, and the Begg’s test was not statistically significant, suggesting an absence of publication bias (p=0.58).


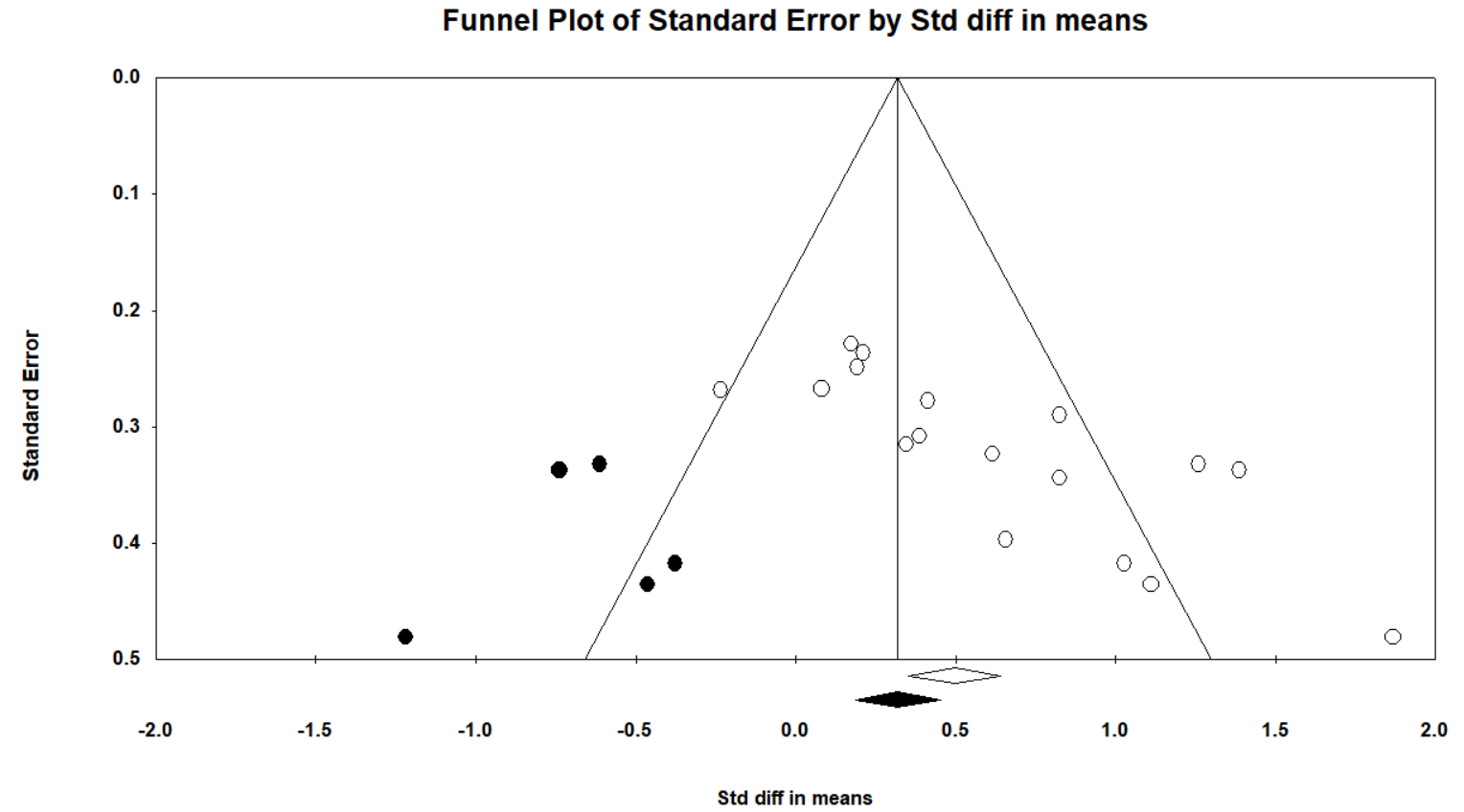


**Supplementary Figure 8**. Funnel plot for gait speed using trim and fill. The gait speed is SMD=0.35, 95%CI [0.08, 0.61].
